# Dlgap1 negatively regulates browning of white fat cells through effects on cell proliferation and apoptosis

**DOI:** 10.1186/s12944-020-01230-w

**Published:** 2020-03-13

**Authors:** Ju Zhang, Jie Yang, Nan Yang, Jianfei Ma, Datong Lu, Yanhua Dong, Hao Liang, Dongjun Liu, Ming Cang

**Affiliations:** 1 State Key Laboratory of Reproductive Regulation & Breeding of Grassland Livestock, Inner Mongolia University, Hohhot, 010070 China; 2 College of Life Science, Inner Mongolia University, Hohhot, 010070 China

**Keywords:** Dlgap1, Proliferation, Apoptosis, Brown fat, White fat

## Abstract

**Background:**

Obesity is a metabolic imbalance characterized by excessive deposition of white fat. The browning of white fat can effectively treat obesity and related diseases. Although *Dlgap1* (Discs, Large (Drosophila) Homolog-Associated Protein 1) is suspected to have an effect on this process, no empirical evidence is available.

**Methods:**

To understand the role of *Dlgap1*, we cultured white and brown fat cells, then performed overexpression and knockout experiments.

**Results:**

We found that *Dlgap1* overexpression in brown adipocytes inhibits brown-fat-related gene expression, promotes white-fat-related genes, while also increasing brown-adipocyte proliferation and apoptosis. However, the gene overexpression has no effect on brown adipocyte maturation. Knocking out *Dlgap1* in white fat cells promotes the expression and inhibition of brown-fat-related and white-fat-related genes, respectively. Additionally, the knockout inhibits white fat cell proliferation and apoptosis, while also promoting their maturation.

**Conclusions:**

*Dlgap1* negatively regulates the browning of white adipocytes by influencing cell proliferation and apoptosis.

## Background

Obesity has become a public health crisis because it is a risk factor for cardiovascular disease, type 2 diabetes, hypertension, stroke, and many cancers [1, 2]. Because chronic energy imbalance is the primary cause of most weight gain, nonsurgical therapy must reduce energy intake or increase energy expenditure, or both [3].

Adipocytes (fat cells) form three metabolically distinct categories: white, beige, and brown [4–6]. White adipose tissue (WAT) stores excess energy in the form of triglycerides, while brown adipose tissue (BAT) transfers chemical energy into heat as a defense against hypothermia and obesity [7]. Recent findings using a novel radiodiagnosis technique revealed unexpectedly high BAT activity in adult humans [6, 8–10]. These studies have also identified complex cell differentiation processes leading to the appearance of active brown adipocytes. Data on rodents suggest that brown adipocytes clustered in defined anatomical BAT depots arise from mesenchymal precursor cells common to the myogenic cell lineage [11]. Brown adipocytes of this origin are now called “classical” or “developmentally programmed” [12]. However, brown adipocytes also appear after thermogenic stimuli at anatomical sites corresponding to WAT, in a process called “browning” [12]. Initial studies suggest that browning of WAT can effectively treat obesity and related diseases [13], but more data are needed to clarify this link.

Existing studies have focused on transcription factors [13–16], secreted proteins [17–19], small RNA [20–23], and other variables [24] that affect browning. In this study, we aimed to identify key genes that regulate browning, laying a solid foundation for studying obesity. The *Dlgap1* protein product is also known as synapse-associated protein 90/postsynaptic density 95 (PSD95)-associated protein 1 (SAPAP1)s [25], DLG and PSD95-associated protein (DAP-1) [26], and guanylate-kinase-associated protein (GKAP) [27]. *Dlgap1* is involved in type 2 diabetes [28], the development and maintenance of normal brain function [29], and interaction with the motor protein dynein [30]. This article verifies that *Dlgap1* expression is significantly different in white and brown fat and negatively regulates the browning of white adipocytes by influencing cell proliferation and apoptosis.

## Methods

### Cell isolation and culture

Inguinal WAT and interscapular BAT were washed thoroughly with sterile phosphate-buffered saline (PBS) to remove blood cells. The tissues were then minced and shaken in 0.2% collagenase type II at 37 °C for 1 h. The cells were centrifuged at 1500 *g* for 5 min to remove the supernatant, and then plated on a collagen-coated Petri dish for culturing at 37 °C in 5% CO_2_. The medium was Dulbecco’s modified Eagle’s medium/nutrient mixture F-12 (DMEM/F12), 10% fetal bovine serum (FBS), 100 U penicillin, and 100 mg/mL streptomycin.

### Construction of Dlgap1 overexpression plasmid

C57BL/6 mouse *Dlgap1* cDNA was cloned from brain tissue. The primers were as follows: 5′-ACAGGATCGAGGAAGCCAGA-3′ (forward) and 5′-CGAAGCTCGGTGGAGAAGAT-3′ (reverse). The PCR product and the pIRES2-EGFP vector were digested with SalI and SmaI restriction endonucleases. Next, the *Dlgap1* fragment was ligated into the pIRES2-EGFP vector.

### Construction of Dlgap1 knockout plasmid using CRISPR/Cas9

The *Dlgap1* coding region was obtained and submitted to http://crispr.dbcls.jp/ and http://crispr.mit.edu/ to design four sgRNAs that targeted the first 200 bp of the *Dlgap1* coding sequence. Targeted sequences were synthesized and annealed to form double-stranded DNA that served as templates for evaluating sgRNAs. These templates were ligated to the skeleton vector pCas-Guide-EF1a-GFP via T4 DNA ligase to form the CRISPR/Cas9 knockout vector.

### Liposome-mediated transfection

Adipocytes at 60–70% confluence were transfected using Lipofectamine LTX with PLUS reagent (Invitrogen, Karlsruhe, Germany). Lipofectamine and plasmids were separately diluted in Opti-MEM (Gibco, Munich, Germany). The two solutions were mixed in a 1:1 ratio, incubated for 5 min at 20–25 °C, and added to the cells.

### RT-qPCR

Total RNA was extracted using RNAiso Plus (TaKaRa Bio, Shiga, Japan) following the manufacturer’s protocol, and then treated with gDNA Eraser to eliminate genomic DNA. Next, cDNA was synthesized using Prime Script RT Enzyme Mix I (TaKaRa Bio). Real-time PCR conditions were based on the manufacturer’s protocol in the TaKaRa Real-Time PCR Kit. A 7500 Real-Time PCR system was used for the reactions. Relative expression was determined using the comparative Ct (2^-ΔΔCt^) method (*n* = 3). The primers are listed in Table S1.

### Western blotting

Western blotting was performed as described previously [31] Overexpression and knockout cells were washed with PBS and lysed in ice-cold lysis buffer with a protease inhibitor cocktail (Mammalian Protein Extraction Kit, KW Biotechnology, Beijing, China). Protein concentrations were measured using the BCA Protein Assay Kit (23,225, Thermo, USA). Proteins (10 mg) were separated with SDS polyacrylamide gel electrophoresis (SDS-PAGE), transferred onto polyvinylidene difluoride (PVDF) membranes, and incubated with primary antibodies overnight at 4 °C. Signals were then visualized using the Thermo Scientific Pierce ECL western blotting substrate and the Tanon 5200 (Tanon, Shanghai, China) detection system.

The primary antibodies used were anti-DLGAP1 (bs-12138R, Bioss), anti-UCP1 (Ab10983, Abcam), anti-PRDM16 (63,976, ABclonal), anti-FNDC5 (Ab174833, Abcam), anti-ACC (21923–1-AP, Proteintech), anti-PPARγ (16643–1-AP, Proteintech), anti-ASC1 (Ab70627, Abcam), anti-PSAT1 (10501–1-AP, Proteintech), anti-FABP4 (15872–1-AP, Proteintech), anti-PGC1α (20658–1-AP, Proteintech), anti-FASN (10624–2-AP, Proteintech), anti-LEPTIN (Ab16227, Abcam), anti-GAPDH (Ab9485, Abcam) and anti-α-tubulin (11224–1-AP, Proteintech). The secondary antibody was HRP-conjugated AffiniPure Goat Anti-Rabbit lgG (H + L) (SA00001–2, Proteintech).

### Immunofluorescence staining

Cells were fixed with 4% paraformaldehyde, permeabilized with 0.25% Triton X-100, and stained with primary antibodies (see “Western blotting”) overnight at 4 °C. After two PBS washes, the cells were incubated with fluorescein isothiocyanate-labeled secondary antibodies (ab7080, Abcam) at room temperature for 1 h. For the negative controls, the primary antibodies were replaced with PBS.

### 5-Ethynyl-2′-deoxyuridine (EdU) assays

This cellular proliferation assay involved fluorescence detection using EdU (RuiBo, Guangzhou, China), a thymidine analog that can be incorporated into newly synthesized DNA during cell proliferation. At 24 h post-transfection, adipocytes were incubated with EdU (50 μM) for 2 h, fixed in 4% paraformaldehyde, permeabilized with 0.5% Triton X-100, and labeled with Apollo® fluorescent dye (RuiBo, Guangzhou, China) following the manufacturer’s protocol. The EdU-positive cells were imaged with a confocal microscope (Nikon, Tokyo, Japan) to calculate their percentage.

### Adipogenic differentiation

Cells at 80% confluence were cultured in induction medium (90% DMEM/F12, 10% FBS, 1 μM dexamethasone, 1 μM 1-methyl-3-isobutylxanthine, 1 μM insulin). Two days later, the medium was replaced with differentiation medium (90% DMEM/F12, 10% FBS, 1 μM insulin) for another 2 d. Controls were cultured in DMEM/F12 with 10% FBS. The cells were then fixed in 4% paraformaldehyde for 15 min and stained with Oil-Red working solution for 30 min. The stained cells were washed with 60% isopropanol and imaged. To measure absorbance (550 nm), isopropyl alcohol was added to dissolved the lipid droplets, and the mixture was placed in a microplate reader (Thermo Scientific™).

### Apoptosis measurements

Cells were digested with trypsin to assay apoptosis using the Cell Cycle and Apoptosis Analysis Kit (7Sea Biotech, Shanghai, China). The treated cells were viewed immediately at room temperature with an inverted fluorescence microscope (Nikon TE2000-U, Japan). Flow cytometry was performed using a BD FACScan system (BD Biosciences, Franklin Lakes, NJ, USA). The data were analyzed with FlowJo 7.6 software.

### Statistical analysis

All data are shown as means ± SD from three individual experiments. Between-group differences were determined with the two-tailed Student’s t-test in GraphPad Prism 5 (GraphPad Software, La Jolla, CA, USA). Significance was set at *P* < 0.05.

## Results

### Isolation and identification of white and brown adipocytes in C57BL/6 mice

**Fig. 1:**
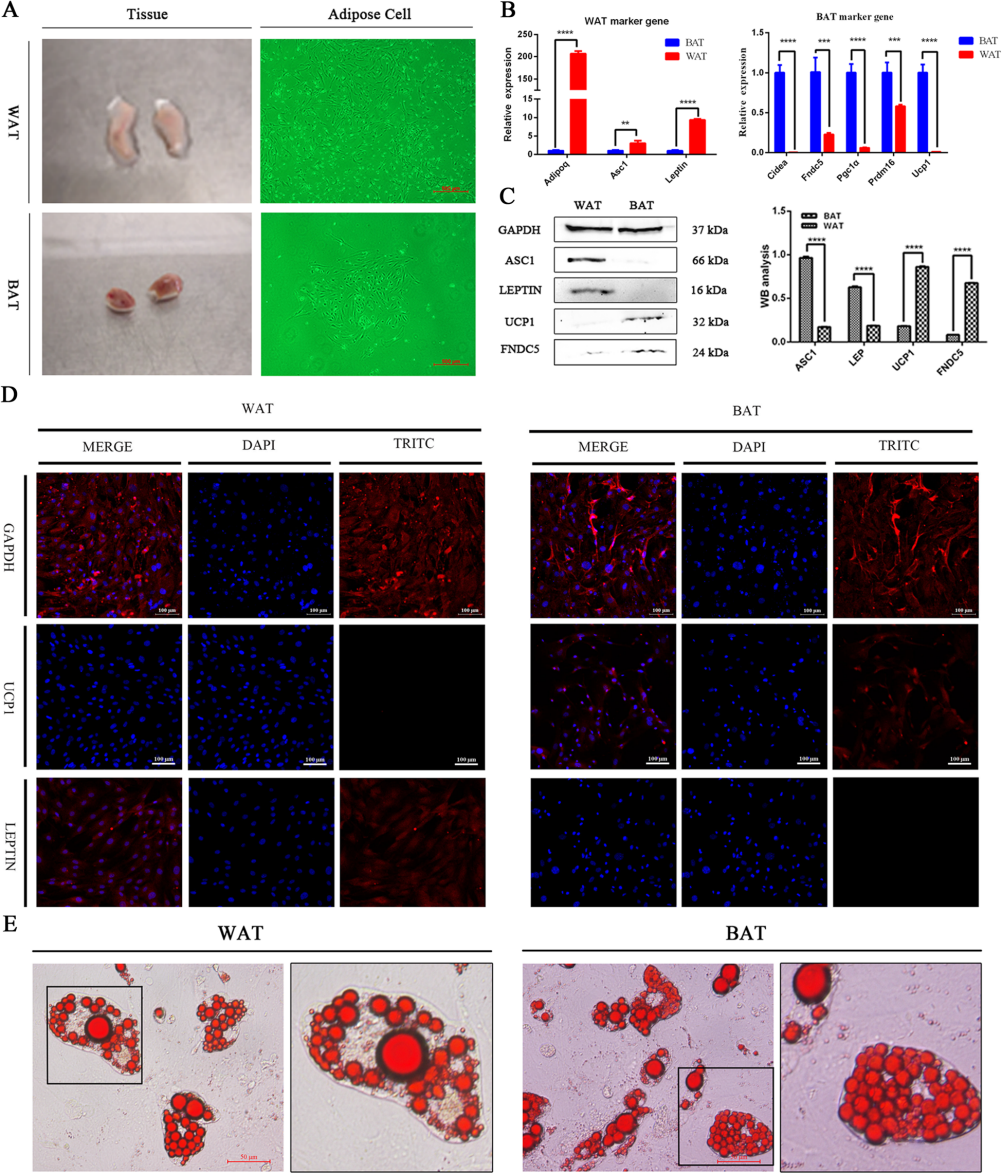
Isolation and characterization of white and brown adipocytes. **a** White fat and brown adipose tissue, left; cultured white and brown adipocytes, right. **b** Results of RT-qPCR on marker genes of white and brown adipocytes. White-fat marker gene, left; brown-fat marker gene, right. **c** Western blots and analysis of gray of white and brown adipocytes in C57BL/6 mice. **d** Immunofluorescence staining of GAPDH (control), LEPTIN, and UCP1 in white and brown adipocytes. Bar represents 100 μm. **e** Morphological observation and Oil Red-O staining of adipogenic differentiation

White adipose tissue accumulates under the skin, mainly in the groin, presenting as an even, milky white color from the large amount of oil present. Brown adipose tissue is located between the shoulder blades and gets its reddish-brown coloration from numerous blood cells (Fig. 1a). After 10 d of culturing experimentally isolated adipocytes, we observed that the white fat cells were 80% confluent, while the brown fat cells were only 40% confluent, indicating a significantly slower growth rate of the latter. Fluorescence phase contrast imaging revealed that the white and brown adipocytes did not differ significantly in morphology (Fig. 1a).

We then analyzed RNA expression of marker genes in white and brown adipocytes using RT-qPCR. We found that the white-fat marker genes *Asc1, Leptin*, and *Adipoq* [32] were 3.03, 9.33, and 207.03 times higher in white adipocytes than in brown adipocytes, respectively. Additionally, the brown-fat marker genes *Cidea, Fndc5, Pgc1α, Prdm16*, and *Ucp1* [10, 33] were expressed at lower levels in white adipocytes, being 0.0034, 0.229, 0.060, 0.582, and 0.0095 times the expression in brown adipocytes, respectively (Fig. 1b). Western blots confirmed the RT-qPCR results (Fig. 1c). Furthermore, immunofluorescence revealed that *Ucp1* expression was nearly absent in white adipocytes, but obvious in brown adipocytes. *Leptin* was expressed in white adipocytes, but not in brown adipocytes (Fig. 1d). Oil red O staining showed that the differentiation of white and brown fat cells occurred at 8 d (Fig. 1e). Cell morphology differed visibly between the two adipocyte types. A mature white adipocyte presented with a large lipid droplet in the center of the cell (single foam fat), whereas a mature brown adipocyte contained many small lipid droplets.

### Overexpression of Dlgap1 reduces browning-specific gene expression

**Fig. 2:**
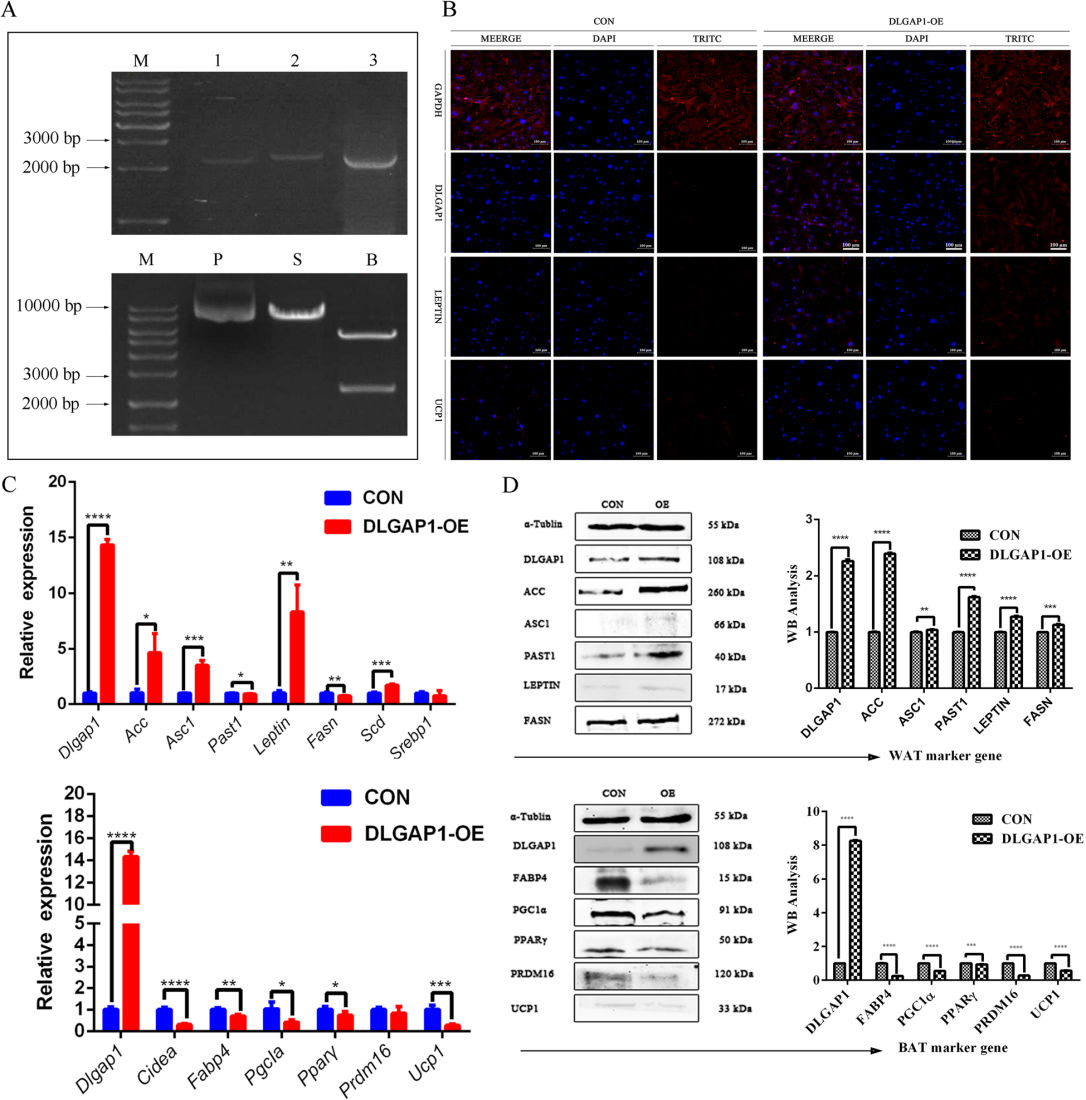
Molecular effects *Dlgap1* overexpression on BAT. **a** Upper panel, electrophoretogram of amplified *Dlgap1* coding sequence; lower panel, identification of the pRIRES2-EGFP-*Dlgap1* plasmid using restriction enzyme digestion. P, plasmid; S, single-enzyme digestion of plasmid; D, double-enzyme digestion of plasmid. **b** Immunofluorescence staining of GAPDH, DLGAP1, LEPTIN, and UCP1 in control and *Dlgap1*-overexpressing BAT. Bar represents 100 μm. **c** Results of RT-qPCR of white- and brown-fat marker genes in control and *Dlgap1*-overexpressing brown fat cells. Top, white-fat marker gene; bottom, brown-fat marker gene. *P < 0.05, ** *P* < 0.01. *** *P* < 0.001, **** *P* < 0.0001. **d** Western blots of *Dlgap1*-overexpressing and control brown adipocytes. Data are means ± SD

Using mouse brain tissue as a template, we amplified the coding sequence of *Dlgap1* and generated the pRIRES2-EGFP-*Dlgap1* overexpression vector (Fig. 2a). The constructed vector was identified through enzyme digestion and electrophoresis (Fig. 2a). The vector’s successful construction was further verified through sequencing.

At 48 h post-transfection of the overexpression vector into brown adipocytes, we analyzed RNA and protein expression. Our RT-qPCR results showed that *Dlgap1* overexpression in brown adipocytes increased *Acc*, *Asc1*, *Leptin* and *Scd* expression, while decreasing *Cidea, Fabp4, Pgc1α, Prdm16, Pparγ* and *Ucp1*expression (Fig. 2c). Western blot assays (Fig. 2d) showed that *Dlgap1* overexpression in brown fat cells dramatically reduced browning-specific proteins (FABP4, PGC1α, *PPARγ,* PRDM16 and UCP1), while increasing white-fat-related proteins (ACC, PAST1, LEPTIN, and FASN). Immunofluorescence analysis of LEPTIN and UCP1 was consistent with the western blot data (Fig. 2b).

### Overexpression of Dlgap1 inhibits brown adipocyte formation

**Fig. 3:**
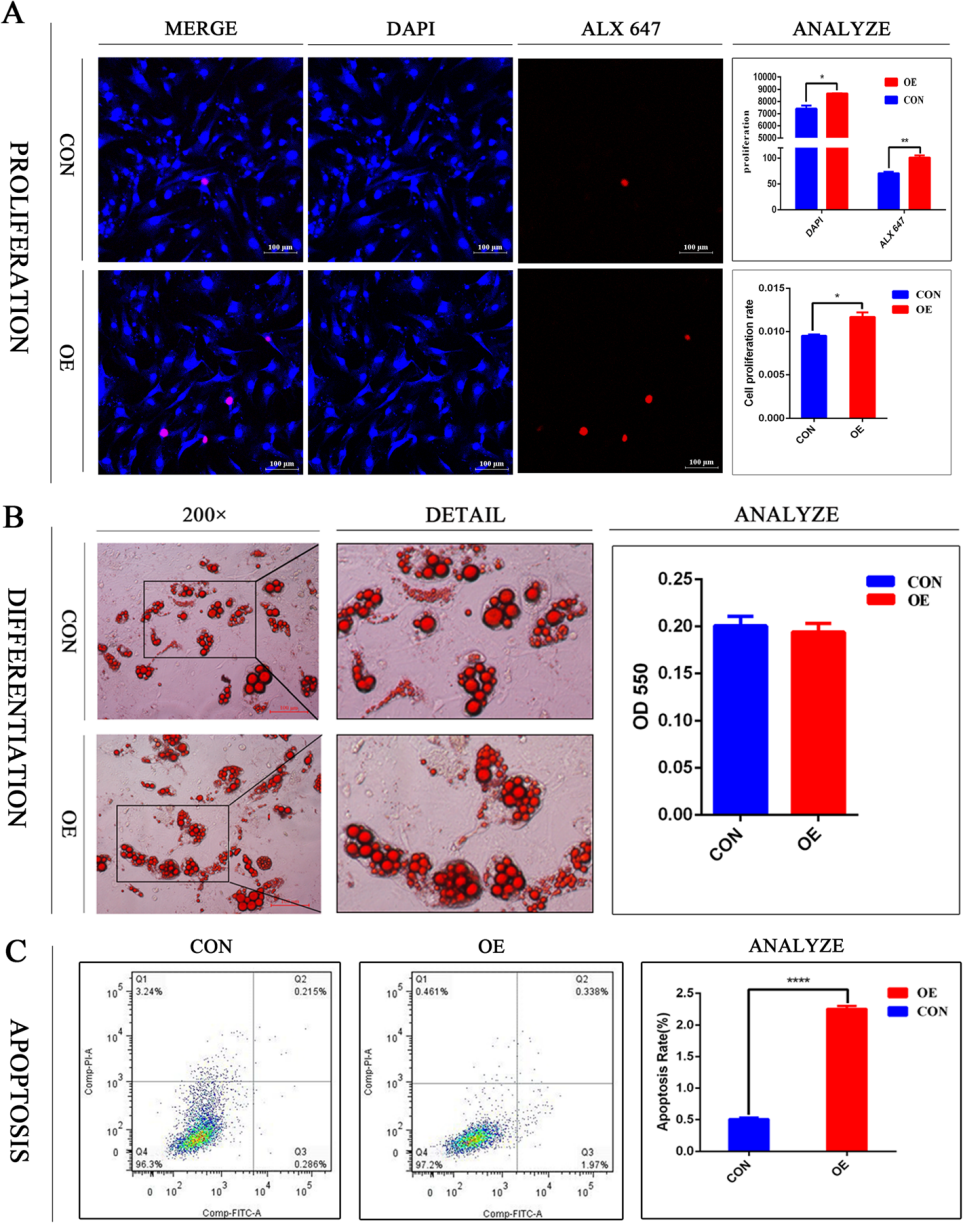
Effect of *Dlgap1* overexpression on BAT cell physiology. **a** Proliferation of *Dlgap1*-overexpressing and control brown adipocytes was detected with EdU assays. Red, EdU staining; blue, cell nuclei stained with Hoechst 33342. **b** Results of Oil Red-O staining of *Dlgap1*-overexpressing and control BAT. Scale =100 μm. Lipid content was measured at 550 nm using a microplate reader. **c** Flow cytometry analysis of apoptosis among *Dlgap1*-overexpressing and control brown adipocytes

The results of EdU proliferation assays revealed that *Dlgap1* overexpression increased brown adipocyte formation. Fluorescent cells were counted under a confocal laser microscope (Fig. 3a). Oil red O staining showed that *Dlgap1* overexpression did not significantly influence adipogenic differentiation (Fig. 3b). Flow cytometry revealed that *Dlgap1* overexpression inhibited brown adipocyte apoptosis. As a result, the experimental overexpression group had 4.6 times more than the control group did (Apoptosis rate = Q2 + Q3) (Fig. 3c). These results indicate that *Dlgap1* is required for the full activation of a gene program favoring brown adipocytes.

### Knocking out Dlgap1 promotes browning-specific gene expression

**Fig. 4:**
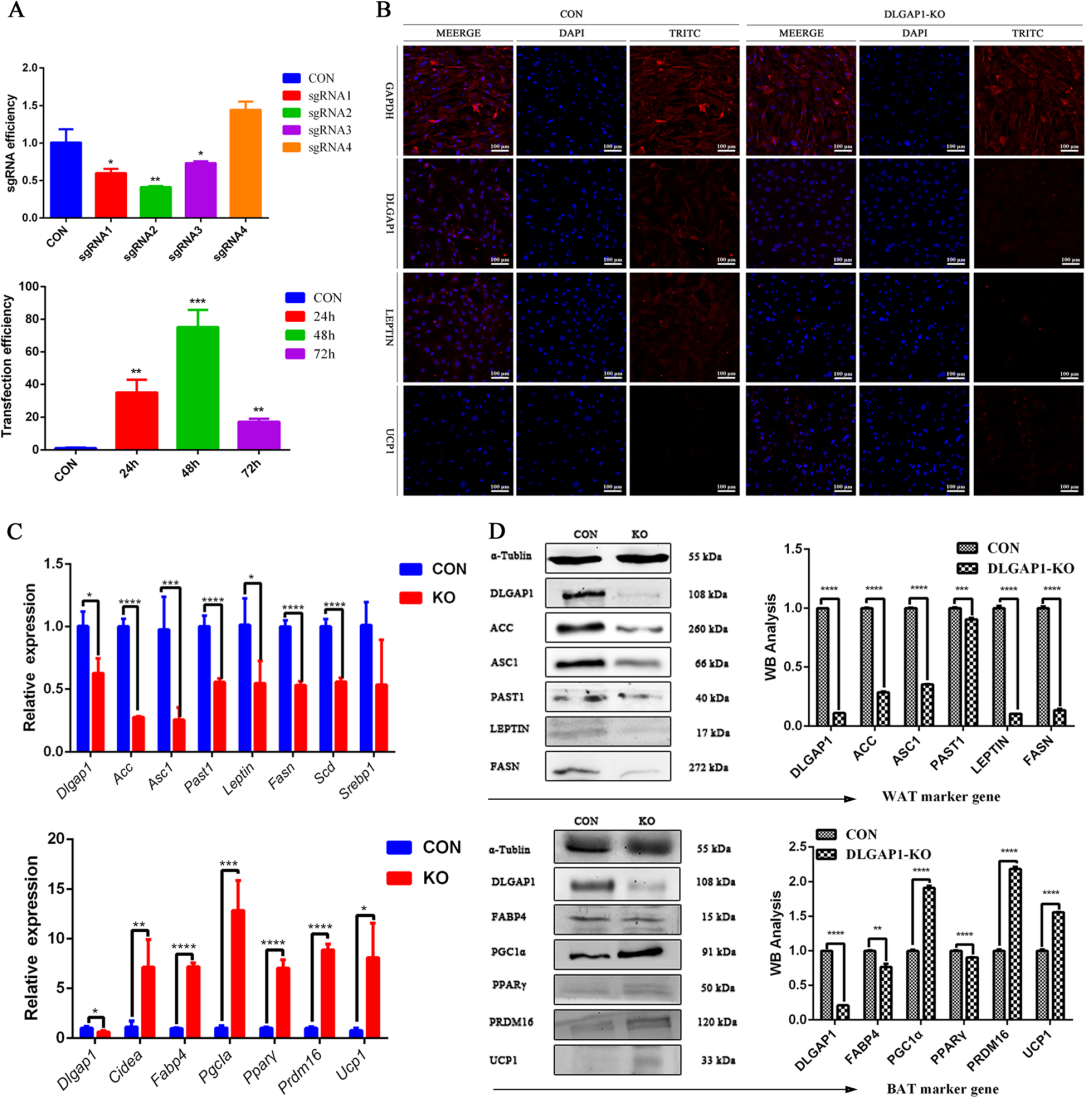
Molecular effects of *Dlgap1* knockout on white adipocytes. **a** Upper panel, sgRNA efficiency test; lower panel, cell transfection efficiency test after 24, 48, and 72 h. **b** Immunofluorescence staining of GAPDH, DLGAP1, LEPTIN, and UCP1 in control and *Dlgap1*-knockout white adipocytes. Bar represents 100 μm. **c** Results of RT-qPCR of white and brown-fat marker genes in control and *Dlgap1*-knockout white adipocytes. Top, white-fat marker gene; bottom, brown-fat marker gene. *P < 0.05, ** *P* < 0.01. *** *P* < 0.001, **** *P* < 0.0001. **d** Western blots of proteins in *Dlgap1-*knockout and control cells. Data are means ± SD

To knock out *Dlgap1* using CRISPR/Cas9 technology, we designed four sgRNA pairs and determined their cutting efficiency. We found that sgRNA1, sgRNA2, and sgRNA3 acted on adipocytes, with sgRNA2 exhibiting the highest cutting efficiency (Fig. 4a). We also investigated how different transfection times affected transfection efficiency, and demonstrated that 48 h post-transfection was optimal (Fig. 4a).

After generating *Dlgap1-*knockout white adipocytes, our RT-qPCR analysis of fat marker genes showed that *Acc, Asc1, Past1, Leptin, Fasn,* and *Scd* expression decreased, while *Cidea, Fabp4, Pgc1α, Pparγ, Prdm16*, and *Ucp1*were upregulated (Fig. 4c). Western blots confirmed *Dlgap1* downregulation, along with decreases in white-fat marker proteins (ACC, ASC1, PAST1, LEPTIN and FASN) to different degrees and increases in brown-fat markers (PGC1α, PRDM16, and UCP1) (Fig. 4d). Consistent with the western blot data, immunofluorescence showed a decrease in LEPTIN expression and increase in UCP1 expression (Fig. 4b). Taken together, these results suggest that *Dlgap1* knockout in white adipocytes promotes browning.

### Knocking out Dlgap1 affects white adipocyte formation

**Fig. 5:**
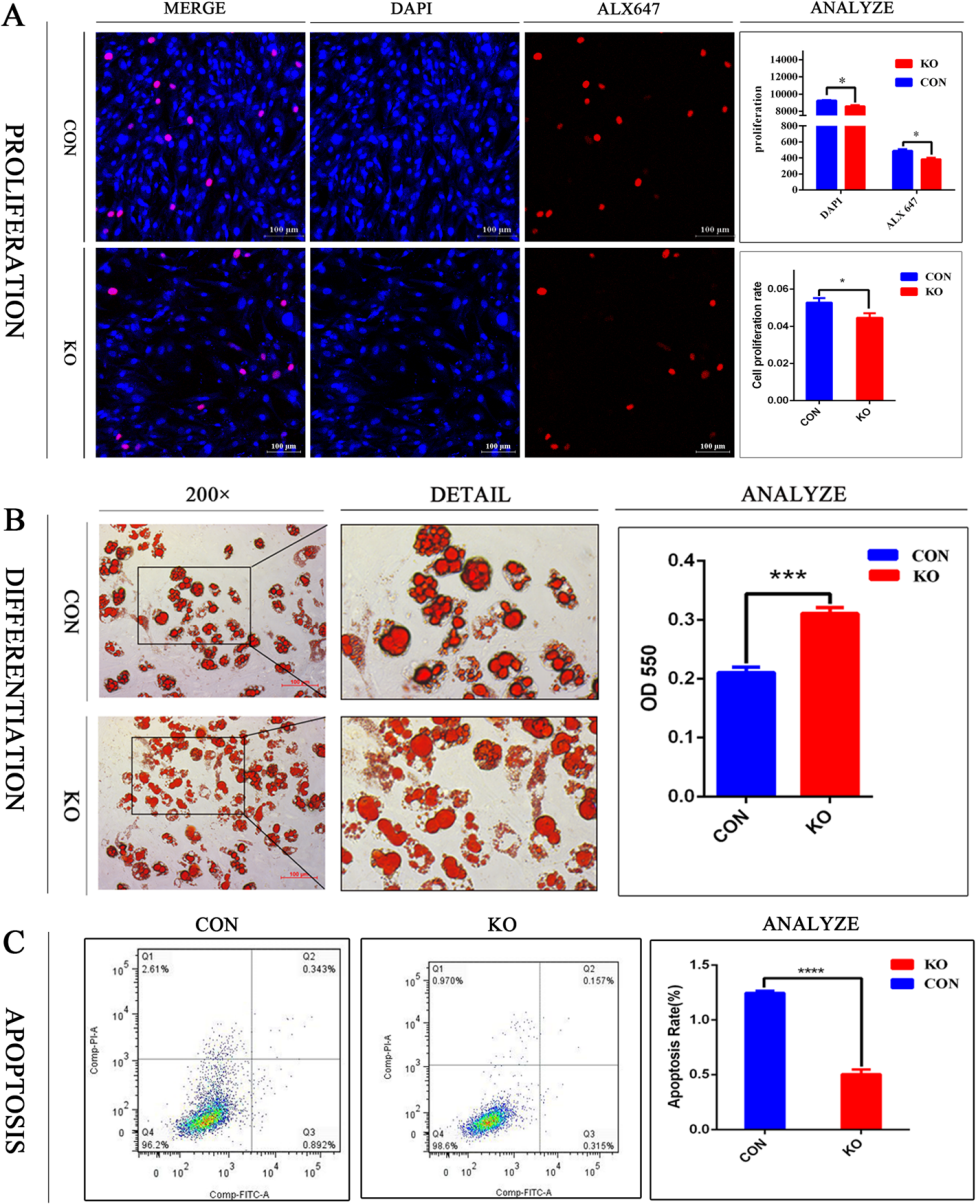
Effects of *Dlgap1* knockout on the cell physiology of white adipocytes. **a** Proliferation of *Dlgap1*-knockout and control cells was detected by EdU. Red, EdU staining; blue, cell nuclei stained with Hoechst 33342. **b** Oil Red-O staining of *Dlgap1*-knockout and control cells. Scale =100 μm. Lipid content was measured at 550 nm using a microplate reader. **c** Flow cytometry analysis of apoptosis among *Dlgap1*-knockout and control cells

The results of EdU assays indicated that *Dlgap1* knockout in white adipocytes changed proliferation by 0.85 times compared with that of the control group (Fig. 5a). Oil red O staining indicated that *Dlgap1* knockout increased adipogenic differentiation (Fig. 5b). Flow cytometry then showed that *Dlgap1* knockout slowed adipocyte apoptosis. Furthermore, the knockout group had 0.38 times apoptosis rate as the control group did (Fig. 5c).

## Discussion

White adipose tissue has emerged as a key determinant of healthy metabolism and metabolic dysfunction. This realization is paralleled by the finding that adult humans have heat-dissipating BAT, an important contributor to energy balance and a possible therapeutic target for treating metabolic disease [34]. We propose that successful targeting of brown and white adipose tissues will depend on research that elucidates their developmental cell-type-specific functional regulators. Here, we explored the effect of *Dlgap1* on white fat browning and revealed that *Dlgap1* is a negative regulator of this process by influencing cell proliferation and apoptosis.

Genetic *Dlgap1* variants are associated with neuropsychiatric disorders, including schizophrenia [35], autism spectrum disorder [36], and obsessive-compulsive disorder [37]. However, the mechanism of *Dlgap1* in the browning of white fat remains poorly understood. Certain *Dlgap1* genetic variants are underrepresented in patients with type 2 diabetes in the Netherlands and Korea [28, 38]. Because both obesity and type 2 diabetes are characterized by imbalances in energy metabolism in the human body, *Dlgap1* may play an unidentified common role in both conditions. Additionally, *Dlgap1* functional variants or genetic variants of neighboring genes that have strong linkage disequilibrium with *Dlgap1* could be responsible for both browning and type 2 diabetes.

At the molecular level, we detected the effect of the *Dlgap1* gene on white and brown fat marker genes by RT-qPCR, western blots, and immunofluorescence, and the results of the three methods were consistent with each other. The *Dlgap1* gene was significantly differentially expressed in white and brown fat cells. Knockout of *Dlgap1* increased BAT-specific gene expression, while overexpression of *Dlgap1* reduced BAT-specific gene expression, especially the expression of UCP1. UCP1 protein plays a key role in the mitochondria in brown fat cells to increase heat production. Viral delivery of irisin, which caused only a moderate increase (~ 3-fold) in circulating levels, was shown previously to stimulate a 10–20-fold increase in UCP1 levels, increase energy expenditure, and improve glucose tolerance in high-fat fed mice [39].

At the cell level, we examined the effects of *Dlgap1* on adipocyte proliferation, differentiation, and apoptosis. We found that *Dlgap1* knockout inhibits white adipocyte proliferation, while its overexpression increases dipocyte proliferation. Relevant studies have shown that PPARγ is highly expressed in adipose tissue and is a key factor regulating adipocyte proliferation, differentiation, and increased insulin sensitivity [40]. The mechanisms underlying these patterns may be that *Dlgap1* expression affects PPARγ, peroxisome proliferator-activated receptor gamma, which also inhibits cell proliferation. In summary, we successfully constructed the overexpression and knockout vector of the *Dlgap1* gene, and verified that *Dlgap1* negatively regulates the browning of white fat by affecting cell proliferation and differentiation. This study identifies a new function of the *Dlgap1* gene in regulating the browning of white fat, laying a foundation for the alleviation and treatment of obesity.

## Conclusions

1. *Dlgap1* overexpression in brown adipocytes inhibits brown-fat-related gene expression and promotes white-fat-related gene expression, while also increasing brown adipocyte proliferation and apoptosis.
2. Knocking out *Dlgap1* in white fat cells promotes the expression and inhibition of brown-fat-related and white-fat-related genes, respectively. Additionally, knockout inhibits white fat cell proliferation and apoptosis.
3. *Dlgap1* negatively regulates the browning of white adipocytes by influencing cell proliferation and apoptosis.

## Supplementary information

**Additional file 1: Table S1.** The primers of q-PCR.

## Data Availability

The datasets used and/or analyzed during the current study are available from the corresponding author on reasonable request.

## Abbreviations

ACC: Acetyl-CoA Carboxylase Alpha
ASC1: Solute Carrier Family 7 Member 10
BAT: Brown Adipose Tissue
CRISPR/Cas9: Clustered Regularly Interspaced Short Palindromic Repeats Cas9
DLGAP1: Discs, Large (Drosophila) Homolog-Associated Protein 1
FABP4: Fatty Acid Binding Protein 4, Adipocyte
FASN: Fatty Acid Synthase
PGC1α: Peroxisome Proliferator-Activated Receptor Gamma Coactivator-1α
PPARγ: Peroxisome Proliferator-Activated Receptor Gamma
PRDM16: Pr Domain Containing 16
PSAT1: Phosphoserine Aminotransferase 1
SCD: Stearoyl-Coa Desaturase (Delta-9-Desaturase)
UCP1: Uncoupling Protein 1
