# Supplementary material for: Dlgap1 negatively regulates browning of white fat cells through effects on cell proliferation and apoptosis
Source: Lipids Health Dis. 2020 Mar 13;19:39. doi: 10.1186/s12944-020-01230-w (PMC7068870; doi:10.1186/s12944-020-01230-w)
Supplement: Supplementary file 1 — Additional file 1: Table S1. The primers of q-PCR. [file 12944_2020_1230_MOESM1_ESM.docx]

Table S1. The primers of q-PCR

| Gene | Primers(5^，^-3^，^) |
| --- | --- |
| *Dlgap1* | Sense: ATCACAGCCCAGAGTAGCA |
|  | Anti-sense: GACTTTGGGTGGAGTTGC |
| *Acc* | Sense: CCGTCTGTGATGACTTTGA |
|  | Anti-sense: CTTTCTGGGTTGGGTGA |
| *Fasn* | Sense: CAAGTGTCCACCAACAAGCG |
|  | Anti-sense: GGAGCGCAGGATAGACTCAC |
| *Srebp1* | Sense: TGCGGCTGTTGTCTACCAT |
|  | Anti-sense: GGCATCTGAGAACTCCCTGT |
| *Fabp4* | Sense: TGATGAAGTCACTCCAGAT |
|  | Anti-sense: AACAGGACCACGACAATGA |
| *Asc1* | Sense: TCGTTTGTGAGCAGGAGGGTTC |
|  | Anti-sense: TGGGTCCTTCGGATACTCGTTC |
| *Past1* | Sense:TACCGCCTTGTCAAGAAACC |
|  | Anti-sense:AGTGGAGCGCCAGAATAGAA |
| *Leptin* | Sense:CCAGGATCAATGACATTTCACACAC |
|  | Anti-sense:AGGTCATTGGCTATCTGCAGCAC |
| *Pgc1α* | Sense: CTGACCACAAACGATGACCCTC |
|  | Anti-sense: GACTGCGGTTGTATGGGACT |
| *Pparγ* | Sense: ATTTCCGCTCCGCACTATCC |
|  | Anti-sense: GAACCCTGACGCTTTATCC |
| *Ucp1* | Sense: CACTCAGGATTGGCCTCTACGAC |
|  | Anti-sense:GCTCTGGGCTTGCATTCTACGAC |
| *Prdm16* | Sense: CCTCGCCATGTGTCAGATCAA |
|  | Anti-sense:CTTTCACATGCACCAACAGTTCC |
| *Scd* | Sense: CGCTGGCACATCAACTTCAC |
|  | Anti-sense:AGGAACTCAGAAGCCCAAAGC |
| *Cidea* | Sense: TGACATTCATGGGATTGCAFACTAA |
|  | Anti-sense: TCCAGCACCAGCGTAACCAG |
| *Gapdh* | Sense: CACGATGGAGGGGCCGGACTCATC |
|  | Anti-sense:TAAAGACCTCTATGCCAACACAGT |
